# Tools and methodology to *in silico* phage discovery in freshwater environments

**DOI:** 10.3389/fmicb.2024.1390726

**Published:** 2024-05-31

**Authors:** Carlos Willian Dias Dantas, David Tavares Martins, Wylerson Guimarães Nogueira, Oscar Victor Cardenas Alegria, Rommel Thiago Jucá Ramos

**Affiliations:** ^1^Department of Biochemistry and Immunology, Institute of Biological Sciences, Federal University of Minas Gerais, Belo Horizonte, Minas Gerais, Brazil; ^2^Laboratory of Simulation and Computational Biology — SIMBIC, High Performance Computing Center — CCAD, Federal University of Pará, Belém, Pará, Brazil; ^3^Laboratory of Bioinformatics and Genomics of Microorganisms, Institute of Biological Sciences, Federal University of Pará, Belém, Pará, Brazil

**Keywords:** freshwater, phage, bacteriophage, cyanophage, virome

## Abstract

Freshwater availability is essential, and its maintenance has become an enormous challenge. Due to population growth and climate changes, freshwater sources are becoming scarce, imposing the need for strategies for its reuse. Currently, the constant discharge of waste into water bodies from human activities leads to the dissemination of pathogenic bacteria, negatively impacting water quality from the source to the infrastructure required for treatment, such as the accumulation of biofilms. Current water treatment methods cannot keep pace with bacterial evolution, which increasingly exhibits a profile of multidrug resistance to antibiotics. Furthermore, using more powerful disinfectants may affect the balance of aquatic ecosystems. Therefore, there is a need to explore sustainable ways to control the spreading of pathogenic bacteria. Bacteriophages can infect bacteria and archaea, hijacking their host machinery to favor their replication. They are widely abundant globally and provide a biological alternative to bacterial treatment with antibiotics. In contrast to common disinfectants and antibiotics, bacteriophages are highly specific, minimizing adverse effects on aquatic microbial communities and offering a lower cost–benefit ratio in production compared to antibiotics. However, due to the difficulty involving cultivating and identifying environmental bacteriophages, alternative approaches using NGS metagenomics in combination with some bioinformatic tools can help identify new bacteriophages that can be useful as an alternative treatment against resistant bacteria. In this review, we discuss advances in exploring the virome of freshwater, as well as current applications of bacteriophages in freshwater treatment, along with current challenges and future perspectives.

## 1 Introduction

Freshwater is an indispensable resource for maintaining life on Earth and has been consistently impacted by the increasing anthropogenic influence. Urban and rural expansion around water bodies, coupled with waste disposal from hospitals, water treatment systems, industry, agriculture, and residences, contribute to rivers and lakes becoming hotspots for the proliferation of pathogenic microorganisms (Reddy et al., 2022). Water disinfection methods have become limited due to the growing demand for water reuse and the inefficiency of a significant portion of antibiotics against the spread of antibiotic-resistant bacteria (Mathieu et al., 2019). Therefore, there is a pressing need to explore natural compounds to control multidrug-resistant bacteria, such as bacteriophages.

Bacteriophages (or phages) are the most abundant biological entities globally. They were first described in the early 1900s, and by now, we know they are widespread in the environment, with estimates of ~10^31^ phages present in the biosphere (Twort, 1915; Rohwer and Edwards, 2002). Phages act as natural predators of bacteria and archaea, and exploit host machinery favoring their own replication (Dion et al., 2020). Phages may interact with bacterial or archaeal hosts by transferring genes that might be ecologically relevant, thus favoring the host genetic fitness through horizontal gene transfer (HGT) (Touchon et al., 2017; De Mandal et al., 2021). When associated with their hosts as prophages, phages may introduce auxiliary metabolism genes that potentially enhance host adaptability (Luo et al., 2022). The initial discovery that phages were highly abundant in aquatic samples (Bergh et al., 1989) laid the groundwork for the eventual determination of their pivotal impact on the ecosystem.

The paramount significance of phages arises from the viral shunt phenomenon, wherein organic matter is recycled through the lysis of host cells, driving global-scale biogeochemical cycles (Breitbart et al., 2018). Bacteriophages represent an ecological alternative to the use of antibiotics, with a lower cost–benefit ratio of production, and exhibit high specificity to their hosts, minimizing dysbiosis (Romero-Calle et al., 2019). They have been employed for at least a century in controlling bacterial infections in humans (Rohde et al., 2018), and have recently been advocated for applications in freshwater environments (Naknaen et al., 2021; Ben Saad et al., 2022; Hu et al., 2023).

Phages can be classified into three groups: (1) virulent bacteriophages that solely undergo the lytic cycle, leading to the lysis of the host cell; (2) temperate phages that can suffer lysogenic cycles, remaining dormant within the host cell (prophages) but can be induced to switch to the lytic or chronic cycle; and (3) filamentous phages: go through a chronic cycle in which viral replication occurs without host cell lysis (Chevallereau et al., 2022; Zhang et al., 2022). Lytic phages are the most desirable due to their cell lysis capability and lower risk of horizontal gene transfer.

Classical studies of phages relied on isolation and culture methods for their identification (Hyman, 2019). Currently, with the advancement of culture-independent methodologies such as metagenomics, databases are increasingly enriched with viral data, enabling a more comprehensive understanding at the taxonomic level and potential interactions of phages with their hosts (Santiago-Rodriguez and Hollister, 2023) showing that bioinformatics tools for mining viral data can be a powerful aid in discovering bacteriophages.

This review discusses the identification of phages in freshwater environments, the primary *in silico* tools used for phage data exploration, and types of phage applications in freshwater. We also discuss the possible challenges and future possibilities for the field.

## 2 Identification of phages in freshwater

The metaviromics field (phage metagenomics) essentially is a shotgun metagenomic approach focused on studying the genomes of viral populations from the environment (Hurwitz and Sullivan, 2013; Coutinho et al., 2017; Moon and Cho, 2021), and due to the importance of freshwater bodies as sources of drinking water, recreation, and commerce, more recent studies have dedicated their efforts to freshwater systems (Bruder et al., 2016). Since water chemistry and hydrological factors can contribute to a dynamic environment on a microbial level, likely to be reflected in the indigenous phage populations, the exploration of metagenomic data sampled from freshwater sources from different biomes and places in the world is bound to reveal a plethora of yet unknown and undocumented species of phages (Hayes et al., 2017; Alanazi et al., 2022).

Previous studies have explored how nutrient availability, seasonality, temperature, and human activity influence freshwater viral communities (Bruder et al., 2016). By example, the study of Mohiuddin and Schellhorn (2015) observed that geographic location does not appear to have had a major impact on viral abundance and diversity for two freshwater lakes of the lower Great Lakes region, Lake Ontario and Lake Erie, since the virome composition of both lakes were found to be similar. However, temporal variation in taxonomic composition was observed for both lakes after a year apart sampling.

Another interesting relationship against phage diversity are the possibly related effects of anthropogenic actions on the microbial environment. The study of Green et al., (2015) of the Virginian Lake Matoaka found viral species richness and diversity to be negatively correlated with the level of human activity at the sampling sites, observing the highest levels of diversity and species richness at the main body of the lake, the area least affected by human activity. Another study, conducted by Fancello et al. (2013), observed that the most anthropogenically influenced out of four perennial ponds of the Mauritanian Sahara presented the lowest amount of viral diversity, and higher abundance of heterotrophic microorganisms and human pathogens.

Freshwater viral metagenomics studies also can assist in tackling significant threats to global health, such as the spread of antibiotic resistance. Not only antibiotic resistance genes (ARGs) can spread across different bacterial populations through horizontal gene transfer mediated by bacteriophages, but bacteriophage-carried ARGs are especially threatening due to their prolonged persistence in the environment, fast replication rates, and ability to infect diverse hosts (Brown-Jaque et al., 2015). Moon et al. (2020) explored ARGs recovered from urban surface water viral metagenome data, revealing novel phage-borne antibiotic resistance genes that were also found in bacterial metagenomes, indicating that they were harbored by actively infecting phages. These results suggest that those environmental bacteriophages could act as reservoirs of unknown ARGs that could be widely disseminated via virus-host interactions and illustrate the potential of the viral metagenomics for the discovery of phages involved in spreading antimicrobial resistance on the environment.

In addition, freshwater metagenomic data can also be used to study the viral ecology in the context of other organisms. Chen et al. (2019) investigated and revealed a worldwide distribution of distinct phage genotypes that may infect Fonsibacter, one the most abundant bacterioplankton in freshwater ecosystems, suggesting their substantial role in shaping indigenous microbial communities and potentially significant influence on biogeochemical cycling.

## 3 *In silico* phage mining with bioinformatic tools

Due to the advances in sequencing technologies and in viral databases, we selected some of the currently most used tools developed to analyze the viral community on metaviromic data. A classic virome analysis pipeline include tools for (i) assembly, (ii) viral sequence prediction, (iii) quality check, (iv) annotation, (v) taxonomy classification, (vi) phage-host prediction tools and (vii) viral microdiversity analysis (Table 1), some being also present in general metagenomic studies (steps i, iv and v). They are essential to understand the diversity of viruses and know their function in the environment, and can be used to identify new uncultivated viral genomes (UViGs) (Green et al., 2015; Moon and Cho, 2021; Naknaen et al., 2021).

**Table 1:** Tools available for metagenomic analysis of data for viral identification from environmental data.

Tool name	Metavirome analysis type	Description	Input type	Accessibility—web or tandalone	Citation
IDBA-UD	Assembly	Assembly of fastq reads	Processed fastq files	Standalone	Peng et al. (2012)
Megahit	Assembly	Assembly of fastq reads	Processed fastq files	Standalone	Li et al. (2016)
MetaSpades	Assembly	Assembly of fastq reads	Processed fastq files	Standalone	Nurk et al. (2017)
MetaViralSpades	Assembly	Assembly of fastq reads	Processed fastq files	Standalone	Antipov et al. (2020)
VirSorter	Viral sequence prediction	Predicts viral regions using probabilistic similarity models and referenced-based protein homology searches	Contigs in FASTA	Standalone	Roux et al. (2015)
Prophet	Viral sequence prediction	Predicts viral regions based on similarity searches against own database	Contigs in FASTA	Standalone	Reis-Cunha et al. (2019)
PHASTEST	Viral sequence prediction	Predicts viral regions based on similarity searches against own database	Contigs in Genbank or FASTA	Standalone or Web	Wishart et al. (2023)
MetaPhinder	Viral sequence prediction	Identifies phage sequences in assembled contigs by integrating BLAST matches to several phage genomes in a database.	Contigs in FASTA	Standalone	Jurtz et al. (2016)
VirFinder	Viral sequence prediction	Machine learning method for identification of viral contigs based on K-mer distribution	Contigs in FASTA	Standalone	Ren et al. (2017)
DeepVirFinder	Viral sequence prediction	Uses convolutional neural networks to learn viral genomic signatures and predict if a sequence is viral	Contigs in FASTA	Standalone	Ren et al. (2020)
PPR-Meta	Viral sequence prediction	Utilizes neural network structure (CNN) to predict viral sequences	Contigs in FASTA	Standalone	Fang et al. (2019)
PhaMers	Viral sequence prediction	Utilizes a machine learning model based on k-mer frequencies to identify viral sequences	Contigs in FASTA	Standalone	Deaton et al. (2019)
VirSorter2	Viral sequence prediction	Predicts viral regions based on HMM alignment to database	Contigs in FASTA	Standalone	Guo et al. (2021)
ViralVerify	Viral sequence prediction	Analyses the gene content of a contig through a Naive Bayesian classifier and classifies it as viral/bacterial/uncertain	Contigs in FASTA	Standalone	Antipov et al. (2020)
geNomad	Viral sequence prediction	Uses an hybrid approach with gene content and a deep neural network to identify sequences of plasmids and viruses	Contigs in FASTA	Standalone	Camargo et al. (2023)
Marvel	Viral sequence prediction	Identify viral bins based on a Random Forest machine learning approach	Bins in FASTA	Standalone	Amgarten et al. (2018)
CheckV	Quality check	Estimation of viral completeness by viral sequence comparison to database of complete viral genomes	Contigs in FASTA	Standalone	Nayfach et al. (2021)
ViralComplete	Quality check	Estimate viral completeness using the Naive Bayesian Classifier to compute the similarity of a sequence to known virus from the RefSeq database	Contigs in FASTA	Standalone	Antipov et al. (2020)
DRAM-v	Annotation	Protein-similarity-based pipeline specific for viral functional and metabolic profiling	Contigs in FASTA plus a affi table generated by VirSorter2	Standalone	Shaffer et al. (2020)
PHANOTATE	Annotation	A gene calling annotation tool that treats a phage genome as a network of paths, being ORFs treated as favorable, and overlaps and gaps less favorable, but still possible. These paths are represented as a weighted network of connections graph to find the optimal path.	Contigs in FASTA	Standalone	Mcnair et al. (2019)
VIBRANT	Annotation	Hybrid pipeline that uses protein similarity and machine learning approach to annotate viral sequences	Contigs in FASTA	Standalone	Kieft et al. (2020)
viral-EggNog-Mapper	Annotation	Pipeline that uses orthology assignment approach to annotate eukaryotic or prokaryotic organisms from genome or metagenome samples	Contigs in FASTA	Standalone and Web	Cantalapiedra et al. (2021)
Kraken 2	Read or contig taxonomic classification	Taxonomic classification of microbiome fastq reads or contigs based on k-mer alignment	Fastq or Fasta files	Standalone	Wood et al. (2019)
VContact 2	Contig taxonomic classification	Utilizes whole genome gene-sharing profiles integrating distance-based hierarchical clustering to generate confidence scores for virus classification	FASTA protein file plus a Gene-to-genome mapping table	Standalone	Bin Jang et al. (2019)
MMSeqs 2	Read or contig taxonomic classification	A protein-search-based taxonomy assignment tool that uses a weighted method to assign taxonomic labels	Fastq or Fasta files	Standalone	Steinegger and Söding (2017)
CAT	Contig taxonomic classification	DIAMOND BLASTP homology for contig taxonomic classification	Contigs in FASTA	Standalone	von Meijenfeldt et al. (2019)
PhaGCN	Contig taxonomic classification	Machine-Learning -Based tools that combines the DNA sequence features and protein sequence similarity to assign taxonomic labels	Contigs in FASTA	Standalone	Shang et al. (2021)
VirusTaxo	Contig taxonomic classification	Taxonomic classification tool that provides a framework to organize the population of viruses from metagenomic data.	Contigs in FASTA	Standalone	Raju et al. (2022)
PHIST	Phage-host prediction	Infer virus host relationships based on the number of k-mers shared between their sequences	FASTA file with host sequences and a FASTA file with viral sequences	Standalone	Zielezinski et al. (2022)
CrisprOpenDB pipeline	Phage-host prediction	Predicts virus-hosts relationships by searching for CRISPR spacer matches and uses several criteria to create predictions	Contigs in FASTA	Standalone and Web	Dion et al. (2021)
RaFah	Phage-host prediction	Utilizes Random Forest model to assign phage-host interaction independent from databases	Contigs in FASTA	Standalone	Coutinho et al. (2021)
VirHostMatcher	Phage-host prediction	Assign virus-host relations based on oligonucleotide frequency similarity	FASTA file with host sequences, FASTA file with viral sequences and Host taxonomy table	Standalone	Ahlgren et al. (2017)
IPHoP	Phage-host prediction	A pipeline that combined multiple tools and methods for phage-host prediction	Contigs in FASTA	Standalone	Roux et al. (2023)
Phyloseq	Diversity analysis	Generate microbial diversity statistics	Virus count table, viral taxonomy table, metadata table	Standalone	McMurdie and Holmes (2013)
MicrobiomeAnalyst	Diversity analysis	Generate microbial diversity statistics	Virus count table, viral taxonomy table, metadata table	Standalone and Web	Dhariwal et al. (2017)
Animalcules	Diversity analysis	Generate microbial diversity statistics	Virus count table, viral taxonomy table, metadata table	Standalone	Zhao et al. (2021)
Microeco	Diversity analysis	Generate microbial diversity statistics	Virus count table, viral taxonomy table, metadata table	Standalone	Liu et al. (2021)

In 2017, Roux et al. (2017), identified IDBA-UD (Peng et al., 2012), Megahit (Li et al., 2016), and MetaSpades (Nurk et al., 2017) as the best available options for assembly of viral contigs from short reads. Later on Sutton et al. (2019) analyzed a set of simulated, mocked, and human gut virome with 16 assemblers and identified MetaSpades as the most efficient. However, it showed less effectiveness in reconstructing microdiversity, being more useful to study the mutation rates of the virome. Additionally, although not present in the previous study for being later published, MetaViralSpades (Antipov et al., 2020), a variation of MetaSpades (Nurk et al., 2017), outperformed it in an analysis of 18 real virome data sets, where the contig completeness was superior in 12 cases (Antipov et al., 2020).

After the assembly, a viral sequence prediction analysis can be applied to filter out phages’ host sequences from the metagenomic data. There are three main approaches (Andrade-Martínez et al., 2022) which includes tools that uses protein homology searches to databases: VirSorter (Roux et al., 2015), Prophet (Reis-Cunha et al., 2019), PHASTEST (Wishart et al., 2023), MetaPhinder (Jurtz et al., 2016); machine learning based tools that employs reference-free viral genomic features detection: VirFinder (Ren et al., 2017), DeepVirFinder (Ren et al., 2020), PPR-Meta (Fang et al., 2019), PhaMers (Deaton et al., 2019); and hybrid tools that employ machine learning classification reference based or reference independent: VirSorter2 (Nurk et al., 2017; Guo et al., 2021), ViralVerify (Nurk et al., 2017), geNomad (Camargo et al., 2023), Marvel (Amgarten et al., 2018), and VIBRANT (Kieft et al., 2020) (which can do the steps of identify viral sequences, annotation, and determine genome quality and completeness) (Table 1). Each methodology will have its limitations, and, for machine learning, is related to how updated is the training dataset, the alignment-based tools may also be limited by how updated are the datasets and the difficulty to handle large data. The best approach would be a combination of results from tools that utilize different methodologies for phage sequence prediction (Andrade-Martínez et al., 2022).

Contigs obtained from short-read metagenomic sequencing are normally segmented and it might have misleading information, making it difficult to perform further analysis. To help with this issue, the use of tools such as CheckV (Nayfach et al., 2021), ViralComplete (Antipov et al., 2020), or VIBRANT (Kieft et al., 2020), that identify the completeness and possible host contamination on viral genomes is essential, but yet, still need improvements due to be dependable of the database of virus and the tools used (Green et al., 2015; Sutton et al., 2019). In terms of annotation, some of the most known tools to predict ORFs (Open Reading Frames) are prodigal (Hyatt et al., 2010), Glimmer (Delcher et al., 2007) and GeneMarks (Besemer et al., 2001; Andrade-Martínez et al., 2022), but there are other more specific tools for virus annotations such as VIBRANT (Kieft et al., 2020), viral-Eggnog-mapper (Cantalapiedra et al., 2021), DRAM-v (Shaffer et al., 2020), and PHANOTATE (Mcnair et al., 2019; Table 1). They are suitable for viral annotation and can be applied in manual curation of possible viral false positive results taking into account characteristics such as number of viral and cellular genes hits, bitscores, absence of viral hallmark genes, and presence of plasmid genes (Guo et al., 2021).

For taxonomic classification, it is currently a challenge to find tools that can classify viral sequences under the latest ICTV taxonomy framework, given the high variability, lack of universally conserved genes, and unknown regarding viruses. Kraken 2 (Wood et al., 2019), is a powerful tool for virus taxonomy and identification, and a study performed by Ho et al. (2023) detected a high F1 score of 0.86 in the correct detection of sequences of a viral mock community of characterized viruses. However, it has limited homology to the reference used, so it’s a good option for the identification of known viruses, and when discovery of new viruses is considered, the use of Kraken 2 combined with other tools is advised (Ho et al., 2023). Among the tools that do taxonomy analysis MMSeqs 2 (Steinegger and Söding, 2017), and CAT (von Meijenfeldt et al., 2019), perform protein homology searches to own databases, VContact 2 (Bin Jang et al., 2019), who employs clustering of viral contigs based on shared genes, PhaGCN (Shang et al., 2021), a deep learning classifier based on gene-sharing networks, and VirusTaxo (Raju et al., 2022), that uses a k-mer enrichment database approach (Table 1). All of these tools have customizable databases or the option to retrain their machine-learning models with the latest ICTV taxonomy, which is essential since the ICTV taxonomy is frequently changing (Zhu et al., 2022).

Considering that freshwater environments are expected to have a considerable percentage of new uncultivated viral genomes (UViGs), if a researcher needs to identify its possible host, it is necessary to perform a phage-host prediction. Current methods include mainly similar oligonucleotide frequency (ONF) analysis (VirHostMatcher) (Ahlgren et al., 2017), k-mer similarity (PHIST) (Zielezinski et al., 2022), CRISPR spacer alignment (Dion et al., 2021), and machine learning algorithms (RAFaH) (Coutinho et al., 2021). For researchers new to metavirome analysis it might helpful to use a software that computes the results of other tools such as iPHoP (Roux et al., 2023), which computes the results of six tools utilizing different methodologies and summarizes the putative taxonomy of phage hosts in a table.

The high volume of data produced by the metagenomic studies stimulated the development of tools to simplify the analysis of metagenomic data that also can be applied to metaviromic datasets. Among them, packages such as Phyloseq (McMurdie and Holmes, 2013), MicrobiomeAnalyst (Dhariwal et al., 2017), Animalcules (Zhao et al., 2021), and Microeco (Liu et al., 2021) are some of the most known integrated R packages available (Wen et al., 2023) and offer great set of graphics to support analysis of environmental viruses and their role through metagenomics.

## 4 Applications of phages in freshwater

Safe drinking water is a high demand limited resource that gains more attention in research as water resources get scarcer worldwide, and multi-resistant water-borne pathogens and overall pollution grows as an even bigger threat to society over the years (Mathieu et al., 2019). Approximately one-ninth of the global population reportedly lacks access to safe drinking water (Jassim et al., 2016). Given the capacity of phages to infect bacterial hosts, they have recently been used as novel tools in water pollution control, to monitor and treat fresh and wastewater (Ji et al., 2021).

### 4.1 Bacteriophages as pollution indicators in water

There have been a few applied methods using phages to evaluate water quality as properties indicators to monitor pathogenic bacteria in wastewater. Immobilized phages have been used on an electrode surface as biorecognition elements, through a technique known as electrochemical impedance spectroscopy (EIS), to detect bacteria, such as *E. coli*, *Staphylococcus aureus*, and *Pseudomonas aeruginosa* (Yue et al., 2017; Zhou et al., 2017; Richter et al., 2018). Phages have also been employed as capture elements by other alternative combinations with nanoparticles for bacterial pathogen detection (Richter et al., 2018), and as biomechanisms to assess membrane performance and monitor membrane integrity in water treatment facilities (McMinn et al., 2017; Wu et al., 2017; Dias et al., 2018).

A specific group of bacteriophages named crAssphages have been proposed as potential universal human feces viral indicators in water bodies (Farkas et al., 2019; Mafumo et al., 2023). CrAssphages were described by Dutilh et al. (2014) as the most abundant phages in the human gut virome. Further studies identified that crAssphages are highly specific and abundant to human feces (Sabar et al., 2022), highly prevalent in sewage samples (Stachler et al., 2017), and maintain correlation to the presence of human enteric viruses in water (Jennings et al., 2020). Given the previous characteristics, crAssphages have been preconized in favor of currently used fecal indicator bacteria (FIB), which poorly explain viral pathogen dynamics in water and have low host specificity, making difficult the identification of the source of contamination (Ward et al., 2020; Toribio-Avedillo et al., 2021; Mafumo et al., 2023). CrAssphage applicability has been evaluated in several countries (Crank et al., 2020; Ward et al., 2020; Sangkaew et al., 2021; Nam et al., 2022) and shows promising possibilities for human fecal contamination detection in freshwater.

### 4.2 Bacteriophages in water treatment

Another challenge that greatly affects the operation of wastewater treatment systems is the formation of flocs and sludge bulking by filamentous microorganisms that proliferate excessively that form thick, viscous foams (Aracic et al., 2015). The study conducted by Petrovski et al. (2011a,b) showed how phages that can lyse multiple host bacteria can circumvent the stability of foams. Additionally, Liu et al. (2015) performed tests in a simulated aeration tank system using isolated Gordonia phages, achieving significant reduction in the sludge sedimentation volume. However, all these methods are still experimental as current research still focuses on evaluating and monitoring the behavior of potential phage candidates on wastewater treatment systems (Reisoglu and Aydin, 2023).

Other lines of research have employed phages as low-cost biological control agents to treat specific pathogenic bacteria in sewage. Studies reported the successful inhibition and lysis of drug-resistant *A. baumannii* (Lin et al., 2010), waterborne disease-causing *Vibrio cholerae* (Wei et al., 2011), and dysentery-causing *Shigella* (Jun et al., 2016) through the combination of different phages in co-culture essays. Also, some studies act on the biological control of cyanobacteria, harmful prokaryotes often causing water blooms on green or red tides and producing cyanotoxins, which endanger the surrounding wildlife, aquatic farming animals, threaten human health and can cause tremendous economic losses (Jassim and Limoges, 2013). The strategy in some of those studies is to isolate and employ cyanophages that effectively reduce phycobilisome proteins and destroy the thylakoid structure of cyanobacteria (Gao et al., 2012; Yoshida-Takashima et al., 2012).

However, for both cases, some problems still emerge in the practical application of phage-based biological control, with the emergence of host-resistant mutants, the reduction of cyanophage infectivity caused by sunlight irradiation, and the feasibility of multiple-host approaches are still challenges to be overcome. Nonetheless, phage-based technology also has the advantage of reducing the use of chemical reagents, thus reinforcing the appeal of such strategies and interest in their future development (Mathieu et al., 2019; Ji et al., 2021).

## 5 Current limitations and perspectives

The study of viral sequences in environmental samples is challenging due to the low representativity or fragmentation of DNA in short sequencing data, the high error rate and the large amount of DNA necessary for long-read sequencing (Warwick-Dugdale et al., 2019). As technology advances, improved read length and sequencing quality have partially addressed this issue. This progress has also opened up the opportunity to implement hybrid approaches for sequencing, combining short and long reads that might allow better environmental virus detection, characterization, and understanding of the microdiversity of virus populations (Warwick-Dugdale et al., 2019; Pratama et al., 2021; Andrade-Martínez et al., 2022).

For the identification of phages, common tools employ distinct methods, such as sequence composition, sequence similarity, and machine learning approaches (Titus Brown and Irber, 2016; Fang et al., 2019; Kieft et al., 2020), but there is no standardization for these techniques. Currently, each method yields slightly different results, and phage identification still relies heavily on trial and error usage of software packages. It is crucial that a golden standard be established to ensure the robustness of methodologies and techniques, thereby enhancing the replicability and reliability of phage identification.

An alternative for an assembly-free, culture-independent study of phages is the analysis of the whole genome of phages by using long-read sequencing technologies, like Oxford nanopore or PacBio technologies (Warwick-Dugdale et al., 2019; Zaragoza-Solas et al., 2022). The advantages of this approach are avoiding over-fragmentation of sequencing data and adopting portable sequencing technologies, allowing the researcher to identify phages from natural sources *in situ* (Warwick-Dugdale et al., 2019). This opportunity leads to the study of phages directly from their natural environment, allowing for the identification of phages and the analysis of the samples in real time, which is a significant and desirable feature for the genomic surveillance field (Lisotto et al., 2021).

Most virus databases are derived from uncultivated viral genomes (UViGs) representing >95% of public databases (Roux et al., 2019), leading to another significant problem: most of the phage-host interactions are obtained solely from *in silico* predictions of the study of metagenomes. This lack of lab-studied observations implies the absence of a clear understanding of host-phage dynamics in nature (Coclet and Roux, 2021). In addition to avoiding the intrinsic wet lab biases, such as the identification of false positive or negative viruses due to contamination, the increase of biases related to the process of sample collection, storage, genetic material extraction, purification, and sequencing (Cantalupo and Pipas, 2019). However, the lack of this holistic vision might affect the build of future databases and the scientific interpretations from related results, so it is vital to keep these current limitations presented by bioinformatics tools in mind and apply different combinations of analysis to confirm the identity of phages coming from metagenomic data (Roux et al., 2013, 2019).

## Author contributions

CD: Writing – original draft. DM: Writing – original draft. WN: Writing – original draft. OA: Formal analysis, Supervision, Writing – review & editing. RR: Conceptualization, Formal analysis, Supervision, Writing – review & editing.
